# Automatic Crack Detection and Classification Method for Subway Tunnel Safety Monitoring

**DOI:** 10.3390/s141019307

**Published:** 2014-10-16

**Authors:** Wenyu Zhang, Zhenjiang Zhang, Dapeng Qi, Yun Liu

**Affiliations:** School of Electronic and Information Engineering, Key Laboratory of Communication and Information Systems, Beijing Municipal Commission of Education, Beijing Jiaotong University, Beijing 100044, China; E-Mails: 13120179@bjtu.edu.cn (W.Z.); 13120112@bjtu.edu.cn (D.Q.); liuyun@bjtu.edu.cn (Y.L.)

**Keywords:** crack detection, crack classification, subway tunnel, line scan cameras

## Abstract

Cracks are an important indicator reflecting the safety status of infrastructures. This paper presents an automatic crack detection and classification methodology for subway tunnel safety monitoring. With the application of high-speed complementary metal-oxide-semiconductor (CMOS) industrial cameras, the tunnel surface can be captured and stored in digital images. In a next step, the local dark regions with potential crack defects are segmented from the original gray-scale images by utilizing morphological image processing techniques and thresholding operations. In the feature extraction process, we present a distance histogram based shape descriptor that effectively describes the spatial shape difference between cracks and other irrelevant objects. Along with other features, the classification results successfully remove over 90% misidentified objects. Also, compared with the original gray-scale images, over 90% of the crack length is preserved in the last output binary images. The proposed approach was tested on the safety monitoring for Beijing Subway Line 1. The experimental results revealed the rules of parameter settings and also proved that the proposed approach is effective and efficient for automatic crack detection and classification.

## 1. Introduction

Subways are one of the main ways to release traffic congestion in urban areas. However, due to effects, such as aging, thermal expansion and contraction, human damage, and topographic change, subway tunnels suffer from breaks in their surfaces and internal structures. These split and slender defects are usually called cracks, which directly reflect the health status of tunnel structures. Timely and accurate monitoring information about cracks is necessary for the maintenance of concrete structures and prevention of accidents.

In past years, inspection of cracks has been done manually by careful and experienced inspectors, a method that is subjective and scarcely efficient. Besides, the poor lighting conditions in the tunnels make it hard for inspectors to see cracks from a distance. Therefore, developing an automatic crack detection and classification method is the inevitable way to solve the problem. The work presented herein endeavors to solve the issues with current crack detection and classification practices, and it is developed for achieving high performance in the following three aspects:

- *Detection rate:* it is the most important requirement for the approach, which means the crack detection and classification approach must guarantee that the vast majority of crack length in the original image is detected in the last output results.
- *Detection accuracy:* under the premise of a high crack detection rate, the crack detection accuracy must be acceptable, which means that the misidentified objects should be removed as much as possible.
- *Detection efficiency:* in practical application, thousands of images are collected, making the processing of so many images a huge task. Therefore, the image processing process must be fast and efficient. Algorithms with high computation complexity are not applicable in this situation.

The above three requirements are the principles for developing the automatic crack detection and classification method. First of all, to guarantee high detection rate, the captured tunnel images should be able to present cracks as much as possible, thus the captured images should have acceptable resolutions. The high-speed CMOS line scan cameras [1] are able to shrink both the space required and the cost involved in securing superior speed, reliability, and image quality. Hence, they have been installed in the subway train and have been used as image sensors to perceive the images of tunnel surfaces. With the obtained high-quality images, image processing techniques become the main issue to be solved.

To be efficient, the image processing techniques used here must avoid complex computations as a prerequisite for high detection rate and accuracy. In gray-scale images, cracks present themselves as dark regions with local minimum gray-level components. Morphological image processing operations have an advantage in segmenting relevant structures without complex iterative calculations. For this reason, they can be used to detect local dark regions in the original gray scale image, in which most irrelevant objects with high gray levels or small regions can be easily eliminated by thresholding operations. To automatically separate the cracks, parameter setting involved in the crack segmentation process is an important subject to be studied. Thus, how to find the optimal parameter settings is another issue to be solved, and it can be answered by experimental results from practical applications.

After the crack segmentation process, there may still be many misidentified objects that appear as cracks. In attempting to distinguish between cracks and unexpected irrelevant objects, feature extraction becomes the key problem to be solved. Distance transformation is usually used in shape segmentation, which inspired us to use distance to map spatial shape into a probability sequence by distance. Thus, a shape descriptor called a distance histogram is proposed to perceive the difference between cracks and irregular objects. The standard deviation of the distance histogram is an effective criterion for describing the degree of irregularity of a spatial shape. Along with the standard deviation of the distance histogram, two additional numerical features are used as the basis for classifying the cracks. With a pattern recognition algorithm or a thresholding classification operation, unexpected objects will be removed and cracks will remain. In conclusion, the main contributions of the proposed methodology include the following three aspects:

1) A new practical crack detecting and classifying approach is proposed, and the approach has good performance in detection rate, detection accuracy, and efficiency.
2) The experimental results describe the rules of the parameter settings, which are significant in practical crack detection and classification applications.
3) The approach finds an effective way to describe the degree of irregularity of a spatial shape specifically for crack classification.

The remainder of this paper is organized as follows: Section 2 gives a brief review of the related works; Section 3 presents a detailed description of the different approach stages; In Section 4, a crack quantification method for measuring crack length and width is introduced; experimental results along with the analysis are given in Section 5; Section 6 concludes this paper and refers to future work related to this research.

### 2. Related Work

Currently, line scan cameras based crack inspection systems are the main method used in infrastructure monitoring. In [2], Yu *et al.*, proposed a mobile robot-based inspection system for crack detection in tunnels. The resolution and integrity of the collected images are imperfect since only one Charged Couple Device (CCD) camera can be installed on the inspection robot. In [3], an adaptive road crack detection system by pavement classification is proposed. A vehicle equipped with line scan cameras is used to store the digital images that will be further processed to identify road cracks. The system performs well for road crack detection, but is not suitable for tunnels. Lee *et al.*, developed a line scan camera based tunnel crack inspection system in [4]. The system is not able to capture high quality images for tunnels because it is only equipped with one camera and the light source is conventional high power halogen light. Besides, the detection accuracy is suspicious because no algorithms is used to classify the detected objects. Recently, a laser scanning technique for damage evaluation based on terrestrial 3D light detection and ranging (LiDAR) has been proposed [5,6]. The technique is able to show the original appearances of the objects being monitored. The technique is meaningful for infrastructures with complex structures, but unnecessary for tunnels since its structures are fixed.

Image processing techniques for automatic crack detection and identification are the focus of this paper. A common practice is to use signal processing methods in combination with other techniques to detect cracks or other defects in the structure surface. In [7], four edge-detection algorithms (fast Haar transform (FHT), fast Fourier transform (FFT), Sobel filtering, and Canny filtering) are applied to detect cracks in bridges. An efficiency comparison is provided, and it is concluded that FFT is more reliable than the three other methods. The wavelet transform-based method is also popular in crack identification [8–10]. A common problem is that, in complex environments, detection results of signal processing methods are influenced by unrelated objects or noises, which is an issue that needs to be remedied.

Mathematical morphology is another flexible image processing technique. In [11], cracks in digitized paintings are detected by a top-hat transform, and the misidentified thin dark strokes are excluded by hue and saturation. Still, many irrelevant objects are misidentified as cracks. Miwa *et al.* [12] uses watershed segmentation to find the linear shape of cracks, but the result distorts the original pattern of the cracks. In [13], a morphological image processing and thresholding method is applied to detect and classify pipe defects. While this method is simple and easily implemented, some dark, thin, or small noises are still misidentified as cracks. In [14], a ranged image morphology-based crack detection for steel slabs is presented, in which over 80% of cracks are classified by the automated on-line detection system.

Some other theory-based methods or algorithms, like probabilistic relaxation and adaptive thresholding [15], k-NN classifiers [16], 3-D scene reconstruction [17], high-speed percolation [18], and Principal Component Analysis (PCA) [19] have also been applied to this problem. These solutions each have their own advantages, such as being anti-noise, high efficiency, and obtaining robust detection results, which all merit further investigation and attention.

## 3. Automatic Crack Detection and Classification

### 3.1. Overview

**Figure 1.**
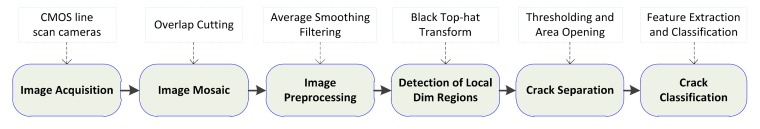
Flowchart of the proposed approach.

The proposed integrated crack detection and classification methodology is divided into six phases, as shown in Figure 1.

The original tunnel images are collected by CMOS line scan cameras. These color images are transferred into gray-scale images for further processing. Firstly the collected images of the nine line scan cameras are stitched together to eliminate overlapping regions. To eliminate the unnecessary local small valleys, an average image-smoothing filter is applied to preprocess the original gray-scale images. In Stage 4, a black top-hat transformation is applied to detect the local dim regions, which contain potential long and dark cracks. Subsequently, objects with low gray levels and large pixel numbers will be segmented by a thresholding operation and morphological area opening. Lastly, the numerical features will be extracted as the input of a classifier, and objects preserved in the final output image will be regarded as cracks.

### 3.2. Image Acquisition

**Figure 2.**
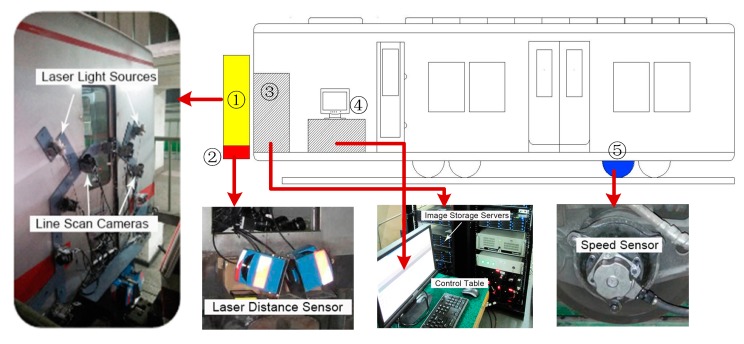
Image acquisition system for subway tunnel monitoring, in which: ① image sensing terminal; ② laser distance sensors; ③ image storage and processing servers; ④ central control system; ⑤ speed sensor.

As shown in Figure 2, the subway crack tunnel image acquisition system is implemented in a modified subway train carriage. The system is consisted by five main parts: image sensing terminal, laser distance sensors, image storage and processing servers, central control system, and speed sensor. The image sensing terminal consists of nine COMS line scan cameras (Basler rall 12288—66 km) and five laser light sources, all of which are anchored by shockproof stationary installations. The nine cameras are placed at different observation angles with a total view angle of 270°. The laser distance sensors are used to measure the distance between the surface and cameras. The speed sensor is planted on the train’s axletree, and the velocity is uploaded to the control system in real time. The collected images are stored in high performance image storage and processing servers. When the train moves forward, the whole system works coherently, the tunnel images will be continuously captured and uploaded to the storage servers.

There are several issues that need to be explained here: (1) the control signals of the nine cameras are synchronized by one serial port controller, hence they capture images at the same time. The nine cameras are always synchronized in capturing images; (2) The train’s unstable movement has little effect on the image quality, because the train’s velocity is always known to the system, the control program is able to adjust the image shooting frequency according the real-time velocity; (3) The light from the carriage has no effect on collected image quality, even though it may cause shadows or unexpected light areas on the tunnel surface. Compared to a laser light source, light leaked from the carriage makes no difference to the image quality. Actually, conventional LED had been used as a light source, but the captured images are nothing but darkness; (4) Since there are so many images, they are all processed offline by the nine high performance computers. For example, In Beijing Subway Line 1, the distance between Xidan Station and Tian’anmen West Station is 1.2 km, and it takes about 38,000 images (each of them is 6144 × 1024, BMP format) captured by the nine cameras.

### 3.3. Image Mosaic

The collected discontiguous images must be stitched together before they are finally presented to the managers. In practice, there is no need to use any image mosaic algorithms to piece these images together, because the horseshoe-shaped subway tunnel has a fixed structure and the covering area of each image is also fixed. Images captured by the same camera can be put together directly. And also, after cutting out the overlap regions, two images of two neighboring cameras fit together directly. Another reason is that we have tried some image mosaic methods, but for several reasons, such as high time consumption and low accuracy, the results are far from satisfactory.

**Figure 3.**
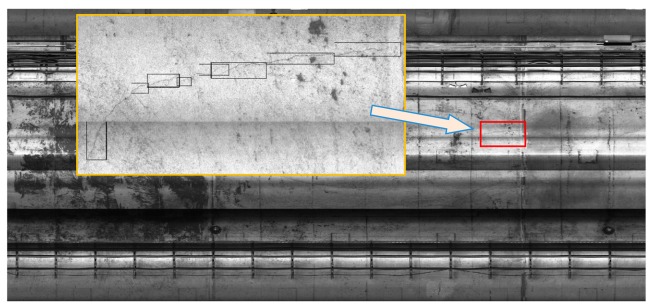
Example of a stitched image.

Figure 3 depicts an example of an image (compressed as low resolution) stitched from 1152 images captured by the nine consecutive cameras. The image stitching method is easily implemented, and the practical results are satisfying. It should be noted that the images are still processed in their original image size, because the obtained stitched image is compressed and the resolution is greatly decreased. The overlapping regions of the images are firstly cut out, and finally the processed images are pieced together after the crack classification process.

### 3.4. Image Preprocessing

**Figure 4.**
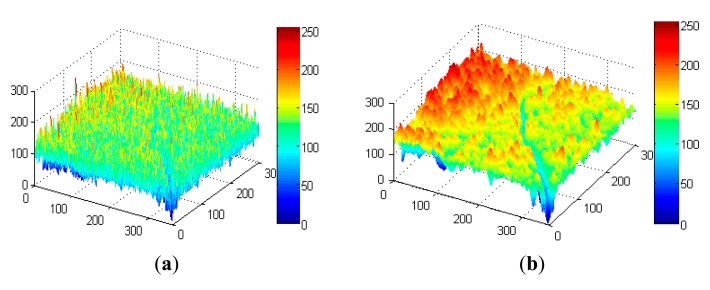
Comparison of original image and smoothed image, with (**a**) original image; (**b**) smoothed image.

The original images have many useless details, especially when in high-resolution. As shown in Figure 4a and b are three-dimensional images of the original image and smoothed image, respectively. In (a), the original image has many needle-like peaks and valleys, which are caused by isolated pixels with high or low gray levels. Since scales of these peaks and valleys are always equal to one or two pixels, an averaging filter will be effective to smooth the original image. If *D* is the applied window matrix, the filtered gray value for pixel *x* is

(1) $$g\left(x\right)=\frac{1}{N_{D}}{\text{∑}}_{x_{n}\in D}g\left(x_{n}\right)$$

where *N_D_* is the pixel number in region *D* and *x_n_* are all the pixels in *D*. *D* can be square or circular, and its window size can be adjusted according to the resolution of the image. A too large window size of *D* will eliminate the details of cracks and a too small window size of *D* is not able to effectively smooth the original image. Therefore, the window size of the average filter should be initialized properly. As shown in (b), the unnecessary peaks and valleys are eliminated by average filtering while preserving the details of cracks. Note that (a) is not specific, because almost every original image is polluted by these salt-pepper like noises. In the following section, positive effects of the average filtering will be illustrated by examples.

### 3.5. Detection of Local Dim Regions

As shown in Figure 4, gray levels of the headstand Y-shape crack are significantly fewer than the other normal regions. In the digitized gray-scale images, cracks are seen as pixels with low luminance components. Morphological operators have an advantage for extracting relevant structures from images captured in complicated environments [20]. Therefore, they are applied to extract the local minimum gray-levels from original image.

Given a gray-scale image *f*, its corresponding black top-hat transformation [21] image can be obtained by

(2) $$f_{BTH}=\phi_{Bn}\left(f\right)-f$$

where *ϕ_Bn_*(*f*) is the closing image set of *f* with respect to the structure element (SE) *B_n_*, which is dilated *n* times from SE *B*_1_. SE *B*_1_ is a flat isotropic structuring set, which can be a disk, square, hexagon, and the like. *B_n_* has the same shape as *B*_1_, but its size increases by *n* compared to SE *B*_1_. *B_n_* can be calculated by

(3) $$B_{n}=\varepsilon_{B}\left(B_{n-1}\right)$$

where *ε_B_* denotes the morphological dilation operation. The number *n* and SE *B* can be set according to the actual situations. Usually, *B*_1_ is simply referred to as *B*. The number *n* has no critical influence on the algorithm’s performance.

### 3.6. Crack Segmentation

After being filtered by the top-hat transformation, the obtained gray-scale sub-graph representation contains potential cracks along with many other objects with local minimum gray-levels. The dark cracks and some other dim regions can be regarded as the objects that are expected to be obtained; other patterns with higher luminance pixels can be seen as the background. Therefore, a thresholding operation is exploited to separate the cracks from the obtained image set.

Let *f_BTH_* be the image filtered by a black top-hat transformation from the original image set *f*. The gray pixel levels are represented from 0 to 255. In a next step, a threshold *T*_0_ of *f_BTH_* is a gray level satisfying

(4) $$w_{0}w_{1}{\left(\mu_{1}-\mu_{0}\right)}^{2}{|}_{l=T_{0}}=\underset{0\leq k\leq 255}{\max}w_{0}w_{1}{\left(\mu_{1}-\mu_{0}\right)}^{2}$$

where *w*_0_, *w*_1_, *μ*_0_ and *μ*_1_ denote the background occurrence probability, objective occurrence probability, background mean levels, and objective mean levels, respectively [22]. The threshold for separating cracks is slightly modified as

(5) $$T=T_{0}+t_{a}$$

where *t_a_* > 0 denotes the acceleration parameter and its unit is a gray level. A suitable value of *t_a_* removes the pixels with gray levels lower than *T*, thus improving the crack detection efficiency. However, an overlarge value of *t_a_* will remove too many pixels with gray levels lower than *T*, decreasing the crack detection accuracy. Hence, it should be initialized properly. The pixel *x* in binary representation of *f_BTH_* can be expressed as

(6) $$f_{T}\left(x\right)=\{\begin{array}{cc} 0 & \text{if}\;x\leq T\\ 1 & \text{if}\;x>T \end{array}$$

**Figure 5.**
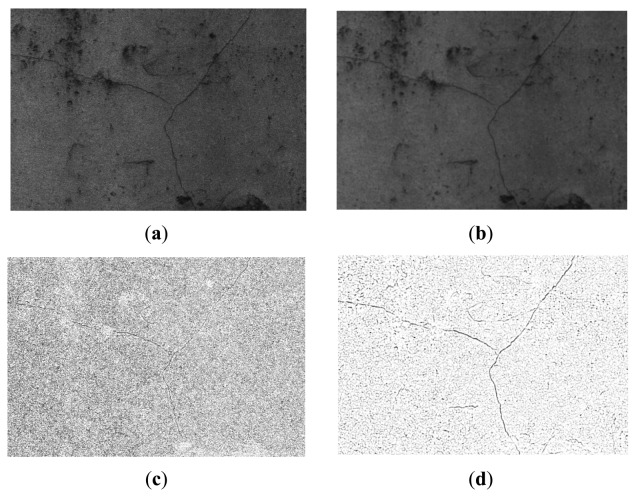
Comparision of the obtained binary images: (a) original gray-scale minage; (**b**) the smoothed image; (**c**) obtained binary image from (**a**); (**d**) obtained binary image from **(b).**

The obtained binary image is expected to give a relatively clear presentation of the cracks compared with the original gray-scale image. Figure 5 is an example illustrating the comparison of the obtained binary images, and its size is 1669 × 1019: (a) is the original gray-scale image, which it contains a Y-shaped crack; (b) is the blurred gray image with an averaging filter window size of four. Their corresponding binary images are (c) and (d), respectively. The binary image (d) obtained from smoothed image (b) clearly represents the cracks. However, cracks in (c) are hard to distinguish. Apparently, average filtering eliminates unnecessary details in the original images, which improves the performance of the crack segmentation process.

**Figure 6.**
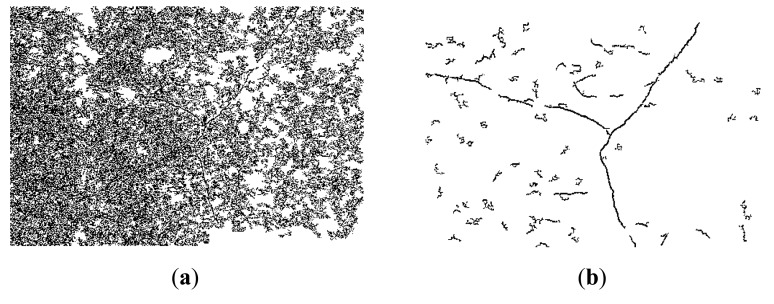

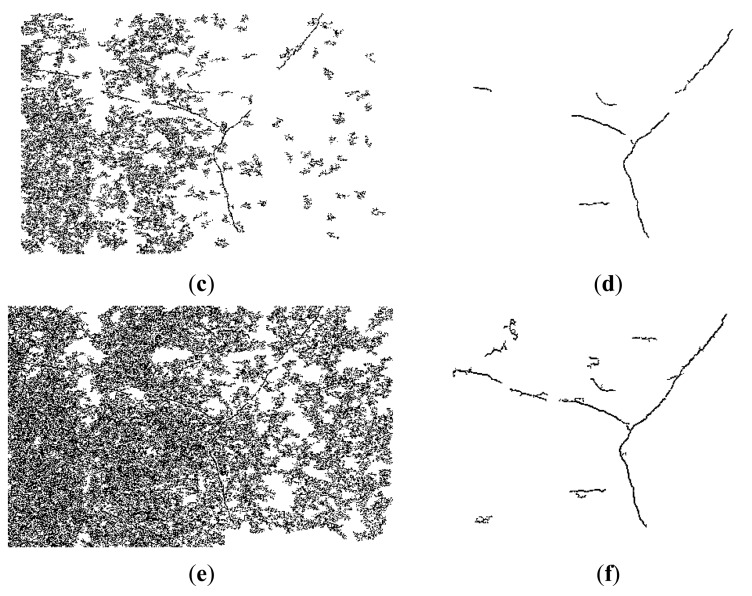
Examples of obtained binary sub-graphs with different parameter settings, *T*_0_ = 47, (**a**) (**b**) *t_a_*=0, *N_B_*=650; (**c**) (**d**) *t_a_*=3, *N_B_*=350; (**e**) (**f**) *t_a_*=0, *N_B_*=650; (**a,c,e**) are results of Figure 5c; (**b,d,f**) are results of Figure 5d.

In Figure 5, many local dark regions or pixels are misidentified as cracks and most of them are noises with small regional sizes. Therefore, a morphological algebraic area opening operation [23], which is an adaptive filter removing the connected components less than a certain number, can be utilized to filter the irrelevant noises. The area opening operation can be expressed as

(7) $$f_{AO,v}\left(X\right)=\{\begin{array}{rr} f_{T}\left(X\right), & \text{if}\;N_{B}\left(X\right)\geq N_{A}\\ \phi , & \text{otherwise} \end{array}$$

where *N_A_* denotes the number threshold value and *X* represents the connected set of *f_T_*. *N_B_*(*X*) is the pixel number of the connected set *X* distributed in SE *B*. *N_A_* can be set according to specific applications, usually it can be set from 300 to 700. Examples of the obtained binary sub-graphs from Figure 5 are shown in Figure 6.

Figure 6 shows the segmentation results of the Y-shaped crack with different parameter settings, (a), (c), and (e) are the binary images obtained from Figure 5c, (b), (d), and (f) are binary images obtained from Figure 5d. Apparently, binary images obtained from the non-smoothed images are heavily polluted by the unnecessary details, which makes cracks hard to be separated using the thresholding operation. Compared with (a), (c), and (e), crack details in (b), (d), and (f) are clearly presented to us. These results prove that the averaging filtering process is able keep the crack details when removing noises from the original images.

For the Y-shape crack, we also have the following observations: In (b), there are still many misidentified objects but the crack details are well preserved; in (d), the detection accuracy increases when *t_a_* increases by three, but the detection rate decreases; compared to (b), detection accuracy is higher in (f) because a larger number threshold *N_B_* removes more misidentified objects. Note that an overly large *N_B_* also decreases the detection rate. These results show that the parameter settings are crucial to the performance of the automatic crack detection approach. In practice, the ideal threshold of *t_a_* is advised as one and *N_B_* is suggested to be set from 400 to 500. Experimental results of how to set these parameters will be presented in the experimental section.

### 3.7. Feature Extraction

After the area opening process, most of the misidentified regions have been removed. However, some large regional irrelevant objects are still preserved as cracks. It is certain that a number threshold *N_A_* large enough will remove these unexpected objects, but some discontinuous parts of cracks will also be filtered, which makes detection rate decrease, which is unacceptable for crack detection.

**Figure 7.**
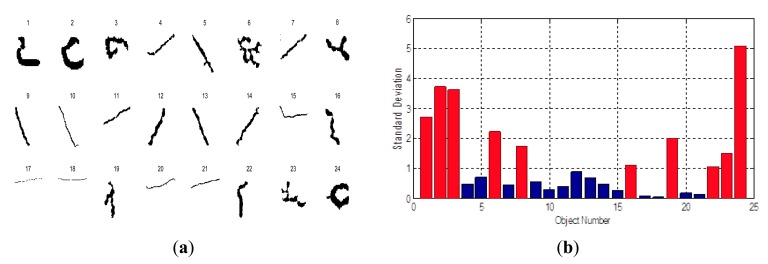
Comparison of the shape distance histogram standard deviation: (**a**) the candidate objects; (**b**) the corresponding standard deviations of distance histograms.

A candidate object is set to belong to two possible classes: cracks and irrelevant irregular objects. Note that some of the crack-like objects with short narrow patterns are also regarded as cracks, because they may be parts of real cracks. In fact, they also may be irrelevant objects that should be deleted. However, to guarantee the detection rate, they must be preserved as cracks. In order to achieve high accuracy of discrimination for crack detection, the irrelevant irregular objects must be removed from the binary image. Since these candidate objects have no fixed shapes, their numerical features should be extracted as the basis of the next classification process. With several experimental verifications, it is concluded that the following three variables are rational and effective in illustrating the differences of the two classes:

*Standard deviation of shape distance histogram:* the spatial shape of an object is an important criterion for classification [24]. From the perspective of visual observation, irrelevant objects are patterns with irregular shapes, while cracks or crack-like objects have slender patterns. A distance histogram-based shape descriptor is presented to measure the irregular degree of a two-dimensional spatial shape:
  In a binary image set, given a connected object *s_c_* with pixels {(*x*_1_, *y*_1_), …, (*x_Nb_, y_Nb_*)}, its central point (*x_c_, y_c_*) can be calculated by
  (8) $$x_{c}=\frac{1}{N_{b}}\sum_{i=1}^{N_{b}}x_{i},y_{c}=\frac{1}{N_{b}}\sum_{i=1}^{N_{b}}y_{i}$$
  with *d_i_* being the rounding Euclidean distance between (*x_i_, y_i_*) and (*x_c_, y_c_*), the shape distance histogram is defined as
  (9) $$p_{i}=\frac{N_{di}}{N_{b}}$$
  where *N_di_* denotes the number of the pixels with the same *d_i_* to the centroid, *p_i_* is the corresponding probability. In this case, the standard deviation of the shape distance histogram can be calculated as
  (10) $$\sigma =\sqrt{\frac{1}{N_{b}}{\left(p_{i}-\mu_{p}\right)}^{2}}$$
  where *μ_p_* is the average distance of all pixels. In general, the distance distribution of irrelevant objects with an irregular shape is heterogeneous. Thus, their standard deviations are larger than those of other classes. The difference of the obtained standard deviations between *ω*_1_ and *ω*_2_ is shown in Figure 7.
  As shown in Figure 7a, the 24 candidate objects are obtained after the previous area opening process. Nos. 1, 2, 3, 6, 8, 16, 19, 22, 23, 24 are irrelevant irregular objects that need to be removed; the rest are cracks. The shape distance histogram standard deviations of the 24 candidate objects are shown in Figure 6b. Note that sizes of these 24 candidate objects have been adjusted for better presentation and the shape distance histogram standard deviations are calculated from their original shapes. The standard deviations of irrelevant objects are all larger than one, which is larger than the objects of the cracks. Apparently, the statistical results show that the standard deviation of shape distance histogram is an effective criterion for crack classification.
*Pixel number:* in the previous image processing stages, the morphological black top-hat transformation is applied to extract local dark areas. However, some regions with uneven surfaces are misidentified as cracks, and they are also isolated from the original gray-scale image. These misidentified areas then become the irrelevant objects. The pixel numbers of these irrelevant objects are always less than those of cracks. The statistical results of 200 candidate objects show that the 21 objects with the largest pixel numbers are all cracks. Therefore, the pixel number is also used as a numerical feature.
*Average gray level:* in the crack segmentation process, the black top-hat transformation separates local dark areas from the gray-scale image, independent of whether they are cracks or not. However, compared to cracks, many local dark regions are bright regions with high gray levels. Some misidentified objects look just like cracks in binary presentation, but they can be classified by their average gray levels in the original images. Therefore, the average gray level of a candidate is also an important feature.

### 3.8. Crack Classification by Extreme Learning Machine Classifier

Subsequently, a pattern recognition algorithm is applied to classify the candidate objects, including the radial basis function neural network (RBFNN) [25], Extreme Learning Machine (ELM) [26], Support Vector Machine (SVM) [27], and K-nearest Neighbors algorithm (KNN) [28]. Let *Γ* = {*τ*_1_,…,*τ_z_*} be the training set with *z* samples and *τ_i_* = (*N_i_, δ_i_, g_i_*), 1 < *i* < *z*, where *N_i_ σ_i_* and *g_i_* are pixel number, standard deviation of shape distance histogram, and average gray level, respectively.

In practice, an ELM is used for both its universal approximation and classification capabilities. And also, it is easy to implement without iterative calculations because its input weights are randomly generated and independent from any specific applications [29]. With adequate training samples, the ELM is able to classify newly observed objects. Given a candidate object, the output is

(11) t=βg(w·τ+b)

where *β*, **w**, and *b* denote the output weight vector, input weight matrix, and bias vector, respectively. **t** is the output vector and **t** = [*t*_1_, *t*_2_]*^T^*. Then the decision can be made by

(12) $$t_{f}=\text{argmax}\left(t_{1},t_{2}\right)$$

If a candidate object belongs to non-cracks, there is no need to keep it. Therefore, the irrelevant irregular objects will be deleted at this stage. After the classification operation, the vast majority of the wrongly identified objects have been removed, but still there are some crack-like objects remaining as cracks. However, to guarantee a high detection rate, these remaining crack-like objects will be preserved in the final outputs.

### 3.9. Crack Classification by Thresholding Classifier

In fact, the classification results obtained by a simple thresholding operation are also acceptable. The sub-graph is obtained by

(13) $$f_{L}\left(X\right)=\{\begin{array}{cc} f_{AO}\left(X\right), & \text{if}\;\sigma \leq T_{\sigma },\bar{g}<T_{g}\\ \phi , & \text{otherwise} \end{array}$$

where *f_L_* (*X*) denotes the sub-graph after thresholding operation, *T_σ_* and *T_g_* are the thresholds of shape distance standard deviation and average gray level, respectively. Note that the pixel number is not used here to avoid removing too many crack-like objects.

**Figure 8.**
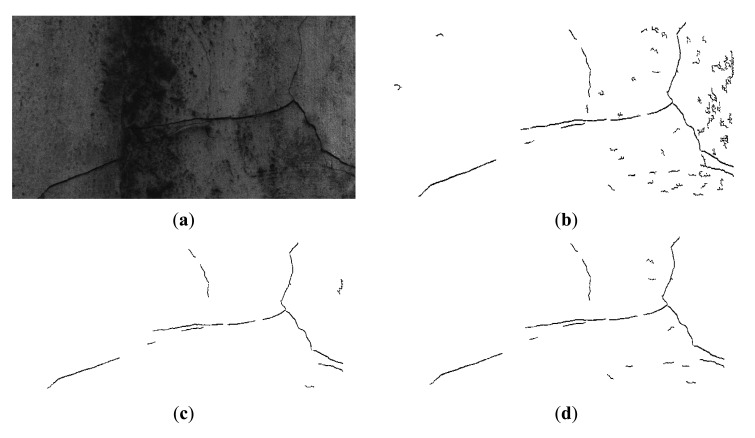
Example of the classified cracks, in which (**a**) represents the original image; (**b**) the detected cracks before classification; (**c**) the classification results by ELM; and (**d**) the classification result by thresholding operation.

Figure 8 shows examples of the classification results: (a) is the original image; (b) is the binary image with candidate objects before classification; (c) is the binary image after classification by ELM; and (d) shows the classification result by thresholding operation. To the human eye, cracks in the original image are easy to distinguish. However, the rough concrete surface is marked by irregularities, protuberances, and ridges, which may be isolated by the morphological black top-hat transformation. As shown in (b), the obtained binary image after area opening has many misidentified irrelevant objects. As shown in (c) and (d), after the classification process, almost all the irrelevant objects in the binary sub-image have been removed. In this example, the detection rates of ELM and thresholding classifier are the same, but the ELM has higher detection accuracy than the thresholding classifier, a finding consistent with most other situations.

## 4. Crack Quantification

**Figure 9.**
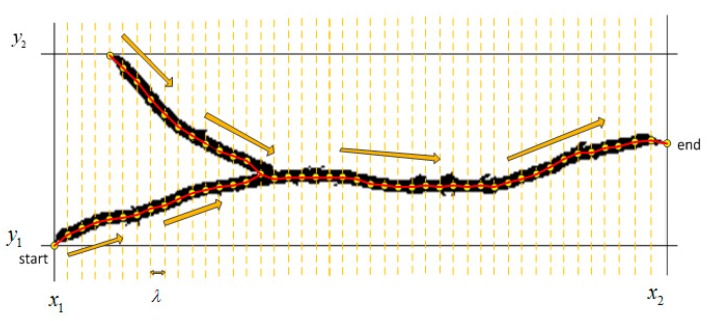
Computation process of crack skeleton graph.

Crack length and crack width are important indicators for evaluating the levels of cracks. The crack length can be calculated by the segmented objects in the final binary image. The crack width can be obtained from the original gray-scale image, as crack locations can be obtained from the final binary image. The quantification algorithm used here is based on the skeleton graph of the crack, as shown in Figure 9.

Firstly the starting point (*x_s_, y_s_*) and the ending point (*x_e_, y_e_*) should be defined. Given a crack with a distribution range ([*x*_1_, *x*_2_], [*y*_1_,*y*_2_]), if *x*_2_ − *x*_1_ > *y*_2_ − *y*_1_, the moving direction is set as *x*_1_ → *x*_2_, the start point and end point are (*x*_1_, *y_s_*) and (*x*_2_, *y_e_*), respectively; if *x*_2_ − *x*_1_ < *y*_2_ − *y*_1_, the moving direction is set as *y*_1_ → *y*_2_, the start point and end point are (*x_s_, y*_1_) and (*x_s_, y*_2_), respectively. Then, the step length *λ is*, is set, a parameter that determines the fitness degree between the skeleton graph and the crack. The skeleton graph can be obtained by moving along step by step in the moving direction. In each step, the mid-point of the corresponding transversal can be calculated, and then the mid-point can be connected with its adjacent point. Supposing there are *N* connection line segments, the mid-points are M = (*m*_1_, …,*m_N_*). Then the crack length can easily be calculated by

(14) $$L_{C}=\rho \overset{N}{\underset{i=1}{\text{∑}}}L_{i}$$

where *L_i_* denotes the length of the *i*th line segment, and *ρ* is the scale of the image. With the skeleton graph, the pixel levels of the mid-points in the original image can be determined.

Suppose *W_ci_* is the crack width with respect to the mid-point *m_i_*. Let *T_w_* be the gray level difference threshold. In the transversal perpendicular to the line segment *m_i_*_−1_ *m_i_*_+1_, pixels with gray levels lower by *T_w_* compared to the gray level of the mid-points will be judged as cracks. Thus the crack width can be obtained by the expression:

(15) $$W_{Ci}=\rho n_{i}$$

where *n_i_* denotes the number of the pixels judged as cracks in the transversal of *m_i_*. Admittedly, the crack quantification is not able to show the real lengths of the cracks when these cracks are detected as discontiguous parts. The accuracy of the crack quantification method with different *λ* and *T_w_* will be presented in the experimental section.

## 5. Experimental Results

To start the experiments, the standard of judging whether a candidate object belongs to cracks or not should be firstly determined. To our knowledge, there is no quantitative definition of cracks in relation to a reference. In this paper, a crack is defined as an object meeting the following characteristics: (1) the object is formed by surface dehiscence; (2) the split width is greater than 0.3 mm; (3) the object length is greater than 15 cm. Any objects with the three characteristics in the surface of the tunnel will be regarded as cracks. Also, the quantitative criteria of crack detection rate and detection accuracy must be defined to measure the performance of the proposed approach. Compared to conventional standards, things are different here because object length is used to calculate the percentages. The crack detection rate and detection accuracy are defined in expressions (16) and (17), respectively.

(16) $$D_{R}=\frac{L_{DC}}{L_{OC}}\times 100\%$$

(17) $$D_{A}=\frac{L_{DC}}{L_{D}}\times 100\%$$

where *L_DC_* and *L_D_* denote the length of detected cracks and length of all detected objects, respectively. *L_DC_* and *L_D_* can be obtained from the automatic detecting results. *L_oc_* represents the length of the original cracks, which is measured by on-the-spot investigation. Before the experiments, a tunnel section (50 m) is determined and its images of lateral surfaces are captured and stored. Then the *D_R_* and *D_A_* are calculated according to the automatic detected results and the on-the-spot inspection results.

In the following three experiments the following will be presented: (1) experimental results on parameter settings; (2) experimental results of accuracy comparison of different classifiers; (3) experimental results on the thresholding classification method; (4) experimental results on the crack quantification method. All of the test images were collected between Xidan Station and Tiananmen West Station, Beijing Subway Line 1. If there are no special instructions, the window size *R_D_*, the structure element size *R_SE_*, the acceleration parameter *T_a_*, and the opening number threshold *N_A_* are set to be 5, 4, 1, and 350, respectively.

### 5.1. Experiments on Different Parameter Settings

**Figure 10.**
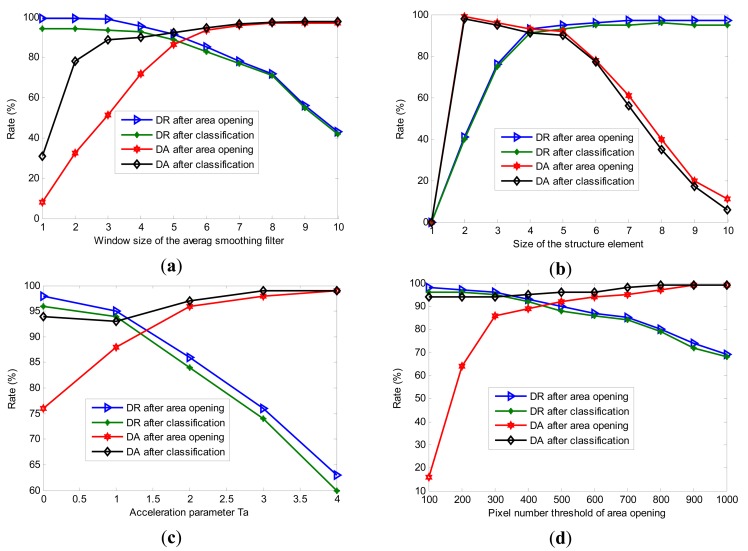
Detection rates and accuracies with different parameters: (**a**) window size of the average smoothing filter; (**b**) size of the structure element; (**c**) acceleration parameter; (**d**) pixel number threshold of area opening.

There are several factors influencing the final detection results, including the average smoothing window size *R_D_*, the structure element size *R_SE_*, the acceleration parameter *T_a_*, and the area opening number threshold *N_A_*. The experimental results of these factors on detection rate and detection accuracy are shown in Figure 10.

Figure 10 shows the detection rate (DR) and detection accuracy (DA) of the obtained images after the area opening and classification process. From Figure 10, the following conclusions can be made:

1) The ideal detection rate (DR) and detection accuracy (DA) are difficult to satisfy at the same time. When increasing the DA, some real cracks will inevitably be removed, which causes a decrease in DR. In fact, it is impossible to achieve the highest DA and DR at the same time. It is advised that the optimal settings of these parameters be set in the range where DA and DR are approximately equal to each other.
2) The results shown in Figure 10 show that the ranges of the parameters should be set as follows: the window size *R_D_*: from three to six; the size of the structure element *R_SE_*: from three to five; the acceleration parameter *T_a_*: at zero or one; and the area opening number threshold *N_A_*: from 300 to 600.

### 5.2. Experiments on Accuracies of Different Classifiers

To verify the performance feature extraction method, different classification algorithms are used to classify the new observed candidate objects. In this test, 200 samples are used as the training set, in which 136 objects belong to the irrelevant object class. The remaining 64 objects are cracks or crack-like objects. The classification algorithms are the Extreme Learning Machine (ELM), radial basis function neural network (RBF), Support Vector Machine (SVM), and K Nearest Neighbors algorithm (KNN). All of the hidden neural nodes of ELM and RBF are 20, the penalty factor *C* of SVM is set as one, and the *K* of the KNN method is set at seven. Given 150 new observed objects, the classification results are shown in Table 1.

As shown in Table 1, over 90% of the candidate objects are correctly classified in the final output results, proving that the proposed method is effective in crack detection and classification. The performances of these classification algorithms show no big differences in practical applications. However, it seems that ELM has stronger discrimination ability than the other classifiers. Besides, compared to other learning-based classifiers, it has a very low computation complexity. Thus, it is advised to use ELM to classify the cracks.

**Figure 11.**
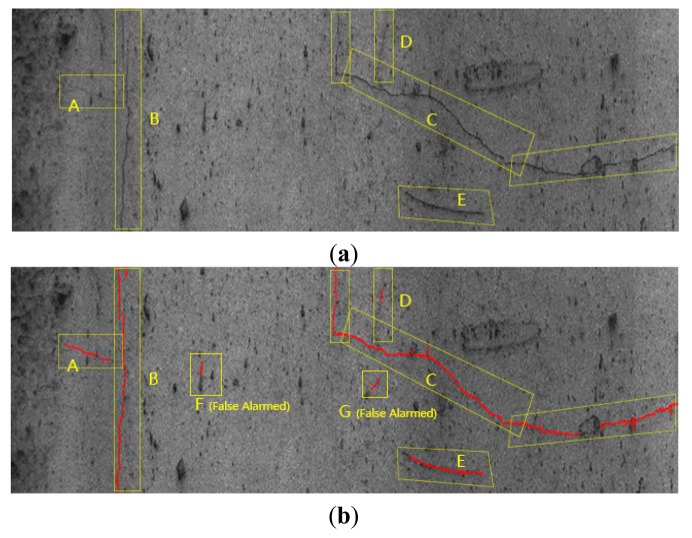

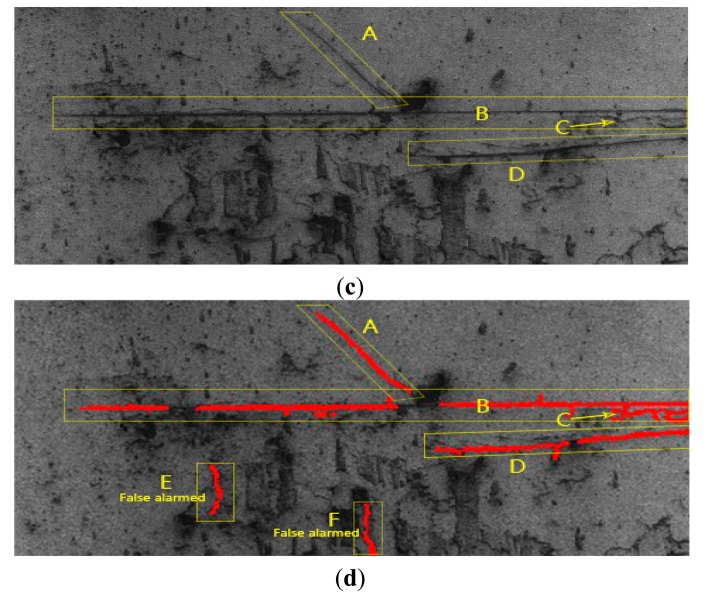
Examples of practical detection results: (**a**) and (**c**) original images with visual detection results; (**b**) and (**d**) automatic detected results of (**a**) and (**c**), respectively.

Figure 11 gives two examples in the practical application using the ELM classifier. The detected cracks are marked in red, whereby (a) and (c) are two original images with visual detection results, and the automatic detected results are shown in (b) and (d), respectively. In general, over 90% crack lengths are correctly detected. However, there are still some misidentified objects. In (a), objects A, B, C, D, and E are cracks determined by visual detection. Figure (d) shows that seven objects are detected as cracks, of which objects F and G are false alarms. Similar problem occurs in (d), objects E and F are also misidentified as cracks. The length percentage of these misidentified objects is no more than 10% compared with the total detected objects.

### 5.3. Experiments on the Thresholding Classifier

**Figure 12.**
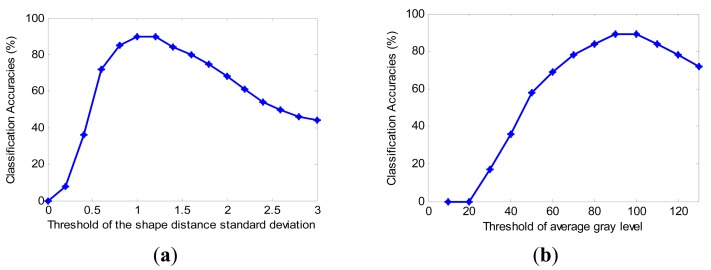
Classification accuracies with different thresholds: (**a**) accuracies with different *T_σ_*; (b) accuracies with different *T_g_*.

As aforementioned, the thresholding operation is also a practical method with which to classify the candidate objects. It is easy to implement without any training. We also studied how to get optimal settings for the thresholding classifier. The classification accuracies with different thresholds are shown in Figure 12.

As shown in Figure 12, the default values of *T_σ_* and *T_g_* are 1.1 and 95, respectively. It is easy to conclude that *T_σ_* should be set from 0.95 to 1.2 and *T_g_* should be set from 95 to 110. The experimental results also show that nearly 90% of cracks are correctly preserved in the last output images. Therefore, the simple thresholding classifier is also an effective way to classify cracks and irrelevant objects.

### 5.4. Experiments on Crack Quantification

**Figure 13.**
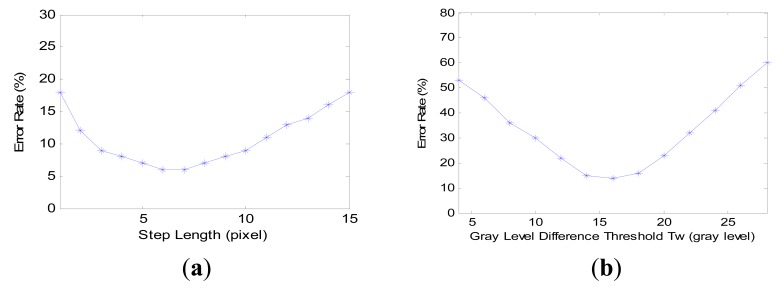
Error rate of the crack quantification method: (**a**) results of length quantification; (**b**) results of width quantification.

To examine the performance of the crack quantification method, 50 samples are used, and all of them have been quantified by on-the-spot investigation. The error rate is calculated by the expression

(18) $$E_{L}=\frac{1}{50}\overset{50}{\underset{i=1}{\text{∑}}}\frac{\left|L_{Di}-L_{oi}\right|}{L_{oi}}\times 100\%$$

where *L_Di_* and *L_oi_* denote the quantified crack length and the on-the-spot measured crack length, respectively. The error rate of crack width is calculated the same way. The scale is 0.15 mm/pixel. The obtained results are shown in Figure 13.

In Figure 13a, the lowest average error rate is 6%, which means the crack length quantification method has a high performance in accuracy. The optimal value range of the step length is 5∼10. However, when cracks are segmented as several parts, the length quantification method is not able to show the real lengths of these cracks. Maybe the problem can be solved by finding a way to correctly stitch the discontiguous parts into a unified one. Compared to crack length quantification, the accuracy of crack width quantification is not that ideal: the lowest average error rate is about 15% when *T_w_* is set from 14 to 18. Mostly, quantified crack lengths are smaller than real crack lengths, especially for the cracks with a width greater than 1.5 mm. The reason is that in digital images obtained by laser scanning, cracks are not able to be presented perfectly as dark pixels, and parts of them are illuminated by the laser lights.

## 6. Conclusions

This paper presents a crack detection and classification approach for subway tunnels on the basis of applying CMOS line scan cameras. A detailed description of the image processing techniques and the optimal parameter settings are given in the experimental section. The proposed approach is easy to implement and effective. The proposed image processing technique for crack detection and classification may be suitable for other state monitoring applications, but not only in subway tunnels. Also, the proposed distance-based shape descriptor may be suitable for other pattern recognition applications. However, it should be emphasized that all the experimental results were obtained from images with a resolution of 0.3 mm. Hence, the advised parameter settings may need to be adjusted in other situations. Future work may include the following: (1) developing a crack mosaic algorithm to connect the discontinuous crack parts and then delete the preserved crack like objects; (2) applying the distance histogram-based shape descriptor to other classification applications; (3) finding a better way to preprocess the original gray-scale images to improve the final detection rate and accuracy.

## Figures and Tables

**Table 1.** Accuracy comparison of different classifiers (%).

**Algorithm**	**Training Accuracy**	**Test Accuracy**
ELM	98.5	91.6
RBF	96.5	90.1
SVM	98.0	91.3
KNN	--	88.7
